# A genome-wide methylation study reveals X chromosome and childhood trauma methylation alterations associated with borderline personality disorder

**DOI:** 10.1038/s41398-020-01139-z

**Published:** 2021-01-05

**Authors:** María J. Arranz, Cristina Gallego-Fabrega, Ana Martín-Blanco, Joaquim Soler, Matilde Elices, Elisabet Dominguez-Clavé, Juliana Salazar, Daniel Vega, Laia Briones-Buixassa, Juan Carlos Pascual

**Affiliations:** 1 Fundació Docència i Recerca Mutua Terrassa, Terrassa, Spain; 2 Centro de Investigación Biomédica en Red de Salud Mental (CIBERSAM), Institut de Recerca Biomèdica Sant Pau (IIB-Sant Pau), Barcelona, Spain; 3 Stroke Pharmacogenomics and Genetics Group, Institut de Recerca Biomèdica Sant Pau (IIB-Sant Pau), Barcelona, Spain; 4 Department of Psychiatry, Hospital de la Santa Creu i Sant Pau, Barcelona, Spain; 5 Department of Psychiatry and Forensic Medicine & Institute of Neurosciences, Universitat Autònoma de Barcelona, Bellaterra, Spain; 6 Translational Medical Oncology Laboratory, Institut de Recerca Biomèdica Sant Pau (IIB-Sant Pau), Bellaterra, Spain; 7 Psychiatry and Mental Health Department, Hospital of Igualada, Consorci Sanitari de l’Anoia & Fundació Sanitària d’Igualada, Igualada, Spain

**Keywords:** Clinical genetics, Psychiatric disorders

## Abstract

Borderline personality disorder (BPD) is a severe and highly prevalent psychiatric disorder, more common in females than in males and with notable differences in presentation between genders. Recent studies have shown that epigenetic modifications such as DNA methylation may modulate gene × environment interactions and impact on neurodevelopment. We conducted an epigenome wide study (Illumina Infinium HumanMethylation450k beadchip) in a group of BPD patients with (*N* = 49) and without (*N* = 47) childhood traumas and in a control group (*N* = 44). Results were confirmed in a replication cohort (*N* = 293 BPD patients and *N* = 114 controls) using EpiTYPER assays. Differentially methylated CpG sites were observed in several genes and intragenic regions in the X chromosome (*PQBP1*, *ZNF41*, *RPL10*, cg07810091 and cg24395855) and in chromosome 6 (TAP2). BPD patients showed significantly lower methylation levels in these CpG sites than healthy controls. These differences seemed to be increased by the existence of childhood trauma. Comparisons between BPD patients with childhood trauma and patients and controls without revealed significant differences in four genes (*POU5F1*, *GGT6*, *TNFRSF13C* and *FAM113B*), none of them in the X chromosome. Gene set enrichment analyses revealed that epigenetic alterations were more frequently found in genes controlling oestrogen regulation, neurogenesis and cell differentiation. These results suggest that epigenetic alterations in the X chromosome and oestrogen-regulation genes may contribute to the development of BPD and explain the differences in presentation between genders. Furthermore, childhood trauma events may modulate the magnitude of the epigenetic alterations contributing to BPD.

## Introduction

Borderline personality disorder (BPD) is a severe psychiatric disorder with a prevalence ranging from 0.5 to 5.9%^1,2^. BPD is characterized by distorted sense of self, unstable interpersonal relationships, strong emotional reactions, and self-harming behaviours. It is also associated with severe psychosocial deterioration and high use of mental health resources^3^. BPD may result from the interaction between genetic and stressful environmental factors such as early childhood traumatic experiences^4–6^. BPD is relatively more common in females than in males: about 75% of people with a clinical diagnosis of BPD are females^7,8^. In addition, the disorder is manifested differentially in men and in women, with notable differences in personality traits^9^. However, the reasons behind these gender differences in prevalence and presentation and the biological factors contributing to this disorder are not yet clear.

Twin and family studies have proven the genetic contribution to BPD, with heritability values of 35–67% for BPD^8,10^. Several studies have associated genetic variants in serotonin (TPH1 and 5-HTT) and *COMT* genes with BPD^10^. Variants in *FKBP5* and *CRHR1* genes involved in the regulation of the hypothalamus–pituitary–adrenal (HPA) axis, which is deregulated in psychiatric disorders, were also found associated with BPD risk^11^. Genome-wide association studies have revealed several risk genes (*DPYD*, *PKP4*) shared with other psychiatric conditions such as bipolar disorder, major depression and schizophrenia^12^. Finally, several studies have observed gene–environmental interactions between early life experiences and monoaminergic or HPA axis genes including *SCL6A4*, *COMT*, *BDNF*, *TPH1*, *FKBP5* and *CRHR2* associated with BPD^2,11,13,14^. However, these findings have not been consistently replicated.

Recent studies have shown that epigenetic regulation factors such as DNA methylation may modulate gene × environment interactions and impact on neurodevelopment. Environmental factors such as early life stress and childhood maltreatment alter DNA methylation patterns^15–20^ and potentiate maladaptive behaviours and psychiatric disorders^21–25^. However, relatively few studies have investigated the contribution of epigenetic factors to BPD. In previous studies, we found alterations in methylation patterns of *NR3C1* in BPD patients with and without childhood traumas^26^, in agreement with Perroud and collaborators’ results^27^. Altered methylation patterns of *HTR2A*, *MAOA*, *MAOB* and *COMT* have also been observed in BPD subjects^28^. Finally, a genome-wide methylation study found that BPD and life adverse events were associated with the methylation status of several genes (*IL17RA*, *KCNQ2* and *EFNB1*, among others)^29^.

In summary, there is strong evidence of the genetic and environmental contribution to the aetiology of BPD and epigenetic regulation may act as a modulator of this interaction. The main aim of this study is to discern the epigenetic events contributing to BPD and to investigate the contribution of childhood trauma to those events.

## Materials and methods

### Participants

#### Discovery sample

A total of *N* = 140 subjects including 44 healthy controls and 96 individuals with a BPD diagnosis were recruited for the discovery genome-wide DNA methylation study. Given the higher prevalence of BPD in women and to decrease genetic heterogeneity, all recruited individuals were female of Caucasian origin. *Control sample*: Healthy controls (CTL) with no history of mental illness, drug use or treatment with psychotropic medication were recruited from the local population. Control participants completed self-reported questionnaires designed to discard BPD symptoms or the presence of childhood trauma. *BPD sample*: patients were recruited from the outpatient BPD unit from Hospital de la Santa Creu i Sant Pau. Inclusion criteria: (1) BPD diagnosis according to DSM-IV criteria and assessed through two semi-structured diagnostic interviews, (2) age 18–50 years, (3) no current episode of any Axis I disorder according to DSM-IV criteria, and (4) no severe physical conditions such as organic brain syndrome, neurological disease, or mental deficiency. The following clinical scales were used for the assessment: Structured clinical interview for DSM IV axis II personality disorders (SCID II)^30^, Revised Diagnostic Interview for Borderlines (DIB-R)^31^, McLean Screening Instrument for Borderline Personality Disorder (MSI-BPD), and Childhood Trauma Questionnaire—Short Form (CTQ-SF)^32^. This questionnaire retrospectively assesses childhood abuse and neglect. It evaluates five types of childhood trauma: sexual, physical, and emotional abuse, and physical and emotional neglect. The questionnaire provides an overall rating and a specific score for every subscale (from 5 to 25), as well as cut-off points to classify each trauma according to the severity of the exposure. According to previous studies which have described a positive correlation between the severity and the number of type of maltreatments, and the methylation of several genes^17,27^, we considered a positive history of trauma if severity was at least moderate in two or more subscales (i.e. cut-off scores of 8 or higher for sexual abuse, 10 or higher for physical abuse, 13 or higher for emotional abuse, 10 or higher for physical neglect, and 15 or higher for emotional neglect). According to the CTQ-SF questionnaire, 49 BPD patients fulfilled the criteria for childhood traumatic experiences (BPD+T), whereas 47 did not fulfil the criteria (BPD-T).

#### Replication sample

The replication cohort included 407 subjects (114 CTL and 293 BPD). Healthy controls had no history of mental illness, drug use, or treatment with psychotropic medication. Patients were recruited from the BPD outpatient units of two hospitals (Hospital de la Santa Creu I Sant Pau and the Consorci Sanitari de l’Anoia, Barcelona). The same inclusion criteria were used for the discovery and replication samples. This sample included *N* = 147 patients with available information for childhood traumatic experiences according to the CTQ-SF^32^. A total of *N* = 81 patients (BPD+T) fulfilled the criteria of traumatic experiences in childhood, whereas *N* = 66 patients (BPD-T) did not fulfil the criteria. All patients and controls were assessed by clinical staff experienced in the evaluation of BPD. All participants were also females of Caucasian origin. Almost 90% of BPD+T patients were exposed to moderate to severe emotional abuse and 60% were exposed to sexual abuse. No significant differences in demographics or clinical severity were observed between BPD patients with and without trauma (Supplementary Table 1).

The study was carried out in accordance with the Declaration of Helsinki and was approved by our research ethics committee. Written informed consent was obtained from all participants who did not receive any economic compensation.

### Methylation studies

#### Genome-wide DNA methylation assay, quality control and pre-processing

DNA was extracted from whole blood (Autopure Qiagen) following the manufacturer’s instructions. DNA bisulphite treatment and PCR amplification were performed by means of EpiTech Bisulfite and the PyroMark PCR kits (Qiagen), respectively. Samples were randomly distributed for all phenotypes of interest (www.randomyzer.org). Genome-wide DNAm was assessed using the Infinium HumanMethylation450K BeadChip (Illumina Inc, San Diego, CA). Quality control (QC) metrics (hybridization rate, bisulfate conversion, number of beads per array and background correction) were examined to determine the success of the bisulfite conversion and subsequent array hybridization. Probe filtering was performed by removing those with detection *p* values >0.05, and were not represented by a minimum of three beads on the array, as described elsewhere^33,34^. Samples with poor bisulfite conversion were also removed.

Single-nucleotide polymorphism-related probes^35^ and all multi-hit probes were excluded, as described in the ChAMP Bioconductor package^36^. A set of quantile normalizations was performed using a background adjustment between-array normalization and a dye bias correction, following previous recommendations^34^. Multidimensional scaling, principal components (PC) analyses and singular value decomposition were used to check for unknown population structures, possible batch effects, as well as other technical artefacts. Next, the Reference-Based Method^37^ was used to adjust the data by cell-type proportion, using the *champ.refbase* function. Finally, the Combat function^38^, implemented via the ChAMP Bioconductor package, was performed to correct technical batch effects.

#### EpiTyper assay

High-Resolution Quantitative Methylation Profiling with EpiTYPER^®^ and the MassARRAY^®^ System (Agena Bioscience, San Diego, California), was used to validate the results from the discovery phase. EpiDesigner software for genomic target selection and PCR primer design was used to select top CpG sites from the discovery study suitable to be analysed by EpiTYPER^®^. Two measures were captured for each CpG site and then averaged to avoid off-measurements. Pairs with a standard deviation bigger than 10% were excluded as part of the quality control.

### Statistical analyses

#### Genome-wide DNA methylation study

Methylation levels of each CpG site were expressed as *β*-values, ranging from 0 to 1 (unmethylated and completely methylated, respectively). Differentially methylated CpG sites (DMCs) were calculated using linear models as described in the limma package^39^. Three different analyses were performed. First, DNAm levels were compared between BPD patients and controls (BPD vs. CTL). Second, BPD+T patients were compared to BPD-T patients and controls pooled together (BPD+T vs. CTL & BPD-T). Third, DNAm levels from BPD patients who had experienced childhood trauma were compared with levels from patients without childhood trauma (BPD+T vs. BPD-T). Analyses were adjusted by age, PC, and presence or absence of trauma in the first analysis, and age and PC for the second and third analyses. The two first PCs that accounted for 91% of variability were included in the analyses. Statistical significant values were set at *p* value <10^−07^ as described by Rakyan et al.^40^. Differentially methylated regions (DMRs) were also calculated using the Bumphunter method; statistical significant values were set at *p* value <0.05.

#### Replication study

Methylation levels of CpG sites an CpG units were expressed as discreet proportions ranging from 0 to 1 by intervals of 0,2 (0 = unmethylated and 1 = completely methylated). DMCs were tested using general linear models. As in the genome-wide DNA methylation study, three different analyses were performed: BPD vs. CTL, BPD+T vs. BPD-T & CTL, BPD+T vs. BPD-T, using the same models. The R statistical computing environment (3.6.3 version) was used to perform all statistical analyses and plots.

### Gene set enrichment analyses (GSEA)

The Molecular Signatures Database (MSigDB)^41^ was used to identify enriched gene sets. CpG sites with unadjusted *p* values <10^−03^ were selected from each discovery analysis, resulting in 723 unique genes from BPD vs. CTL analysis, 1603 from BPD+T vs. BPD-T & CTL and 196 genes from BPD+T vs. BPD-T. We evaluated the overlap between our three gene sets with two MSigDB collections, Gene Ontology (GO) biological processes ontology and Hallmark gene sets. Similar analyses were performed with genes mapping statistically significant DMRs.

## Results

### Genome-wide DNA methylation study

After applying quality controls, 140 individuals and 424,616 probes (87.4% of the total probes contained in the HumanMethylation450 BeadChip array) were included in the analyses.

**Fig. 1:**
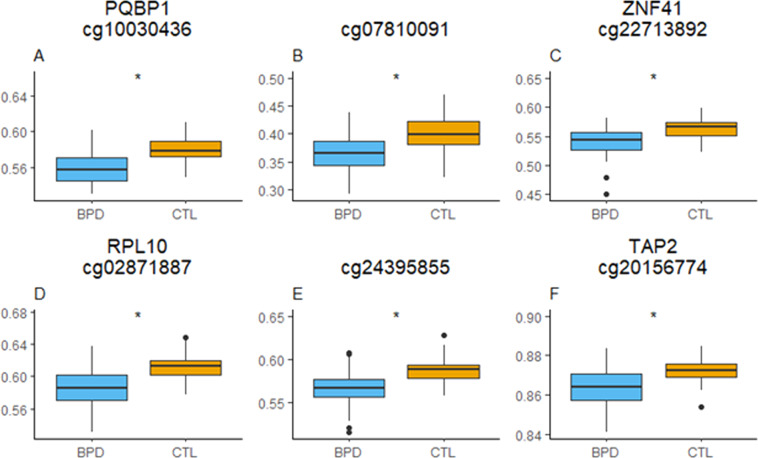
Significantly differentially methylated CpG sites resulting from the comparison between BPD (*N* = 96) and CTL (*N* = 44). *Y*-axis represents methylation levels (*B* values after combat and champ.refbase adjustment, 0 = not methylated, 1 = fully methylated).

The first analysis comparing DNAm levels between BPDs (*N* = 96) and CTL (*N* = 44) showed six CpG sites (*PQBP1* cg10030436, *q* value (FDR adjusted *p* value) = 3.04 × 10^−02^; intergenic cg07810091 *q* value = 3.04 × 10^−02^; *ZNF41* cg22713892, *q* value = 3.15 × 10^−02^; *RPL10* cg02871887, *q* value = 3.15 × 10^−02^; intergenic cg24395855, *q* value = 3.15 × 10^−02^; *TAP2* cg20156774, *q* value = 4.86 × 10^−02^) with differential methylation in our multivariate model (considering age, PC, and presence or absence of childhood trauma as covariates (Table 1). All six CpGs had lower methylation levels in BPD patients than in controls (Fig. 1). In general, the differences in methylation levels with controls were clearer in BPD+T patients than in BPD-T individuals (Supplementary Fig. 1).

**Table 1:** Top 20 differentially methylated CpG sites resulting from the comparison between BPD (*N* = 96) and CTL (*N* = 44) methylation levels.

CpG site	*P* value	*Q* value	*B*	Chr.	Chr. position	Gene	Gene region
**cg10030436**	**1.16E−07**	**3.04E−02**	**5.94**	**X**	**48755633**	***PQBP1***	**5′UTR**^**a**^
**cg07810091**	**1.43E−07**	**3.04E−02**	**5.73**	**X**	**153626455**		**IGR**
**cg22713892**	**3.21E−07**	**3.15E−02**	**4.95**	**X**	**153575581**	***ZNF41***	**5′UTR**
**cg02871887**	**3.31E−07**	**3.15E−02**	**4.92**	**X**	**47518016**	***RPL10***	**TSS200**
**cg24395855**	**3.71E−07**	**3.15E−02**	**4.81**	**X**	**32782796**		**IGR**
**cg20156774**	**6.82E−07**	**4.82E−02**	**4.21**	**6**	**3982903**	***TAP2***	**Body**
cg06753086	1.12E−06	6.29E−02	3.73	1	67430099		IGR
cg11780549	1.19E−06	6.29E−02	3.68	17	48755716	*ASPSCR1*	Body
cg24047905	1.40E−06	6.62E−02	3.51	17	3977385		IGR
cg23390865	1.70E−06	6.70E−02	3.33	X	30174190	*LOC100133957*	TSS1500
cg11435369	1.74E−06	6.70E−02	3.30	7	55670466	*ADAP1*	Body
cg17322683	2.04E−06	7.22E−02	3.15	6	67032472	*RNF39*	3′UTR
cg12054566	2.54E−06	8.31E−02	2.93	X	1510000	*OGT*	1stExon
cg04433201	3.02E−06	8.48E−02	2.77	17	48534493	*FAM101B*	TSS1500
cg25375329	3.10E−06	8.48E−02	2.74	X	942874	*RNF113A*	TSS1500
cg19880947	3.19E−06	8.48E−02	2.71	17	145011888	*SLC43A2*	Body
cg11147309	3.52E−06	8.49E−02	2.62	8	45737011	*PLEC1*	Body
cg11020638	3.63E−06	8.49E−02	2.59	17	1818886	*STAT5A*	Body
cg07478007	3.80E−06	8.49E−02	2.55	1	27188505	*ATAD3B*	Body
cg16061947	4.19E−06	8.89E−02	2.45	11	65550444	*ADRBK1*	TSS1500

*Q* value indicates FDR corrected *p* values. *B* indicates log-odds that the gene is differentially expressed.

Bold letters represent statistically significant results.

^a^CpGs in two studies: BPD vs. CTL and BPD+T vs. BPD-T & CTL.

The second analysis (*N* = 49 BPD+T patients vs. *N* = 91 BPD-T & CTL) revealed five CpGs with lower methylation levels in the BPD+T group (*POU5F1* cg15948871, *q* value = 3.86 × 10^−04^; *GPR55* cg24915915, *q* value = 1.15 × 10^−02^; GGT6 cg04511534, *q* value = 1.15 × 10^−02^; *TNFRSF13C* cg00253346, *q* value= 1.15 × 10^−02^; *FAM113B* cg05478172, *q* value = 2.61 × 10^−02^) after adjusting by age and PC (Table 2 and Supplementary Fig. 2).

**Table 2:** Top 20 differentially methylated CpG sites resulting from the comparison between BPD+T patients (*N* = 49) and BPD-T patients and CTL (*N* = 91).

CpG site	*P* value	*Q* value	*B*	Chr.	Chr. position	Gene	Gene region
**cg15948871**	**9.09E−10**	**3.86E−04**	**10.57**	**6**	**31139620**	**POU5F1**	**TSS1500**^a^
**cg24915915**	**6.01E−08**	**1.15E−02**	**6.45**	**2**	**231799064**	**GPR55**	**IGR**^a^
**cg04511534**	**8.10E−08**	**1.15E−02**	**6.16**	**17**	**4463371**	**GGT6**	**Body**^**a**^
**cg00253346**	**1.09E−07**	**1.16E−02**	**5.87**	**22**	**42324281**	**TNFRSF13C**	**TSS1500**
**cg05478172**	**3.07E−07**	**2.61E−02**	**4.86**	**12**	**47628981**	**FAM113B**	**Body**
cg16465769	7.10E−07	5.03E−02	4.04	6	43266151	SLC22A7	1stExon^a^
cg26529771	1.07E−06	6.20E−02	3.64	16	89260650	CDH15	Body
cg10030436	1.17E−06	6.20E−02	3.56	X	48755633	PQBP1	5′UTR^b^
cg07823755	1.36E−06	6.44E−02	3.40	2	234668355	UGT1A10	Body
cg07583088	1.73E−06	7.25E−02	3.17	16	1181617		IGR
cg20587394	1.88E−06	7.25E−02	3.09	12	54332209	HOXC13	TSS1500
cg13231322	2.21E−06	7.35E−02	2.94	1	32234411		IGR
cg24786705	2.25E−06	7.35E−02	2.92	5	34500318		IGR^a^
cg14153321	2.49E−06	7.49E−02	2.82	22	38246051	EIF3L	Body
cg24073755	2.66E−06	7.49E−02	2.76	1	231955433	DISC2	TSS1500
cg02224755	2.82E−06	7.49E−02	2.70	16	1608969	IFT140	Body
cg14788655	3.41E−06	8.33E−02	2.52	11	40224146	LRRC4C	5′UTR
cg25929552	3.53E−06	8.33E−02	2.48	2	67551782		IGR
cg01619107	3.94E−06	8.57E−02	2.38	12	52515354		IGR
cg10188253	4.22E−06	8.57E−02	2.31	19	43908315	TEX101	5′UTR

*Q* value indicates FDR corrected *p* values. *B* indicates log-odds that the gene is differentially expressed.

Bold letters represent statistically significant results.

^a^CpGs in two studies: BPD+T vs. BPD-T & CTL and BPD+T vs. BPD-T.

^b^CpGs in two studies: BPD vs. CTL and BPD+T vs. BPD-T & CTL.

Comparisons between BPD+T (*N* = 49) and BPD-T (*n* = 47) patients did not show statistically significant results at the 1 × 10^−7^ level. However, 4 of the top 20 CpG findings coincided with top findings in the second analysis (intergenic cg24786705, unadjusted *p* value = 4.11 × 10^−6^; *POU5F1* cg15948871, unadjusted *p* = 1.04 × 10^−5^; *GGT6* cg04511534, unadjusted *p* = 2.02 × 10^−5^; *SLC22A7* cg16465769, unadjusted *p* = 2.75 × 10^−5^ (Table 3 and Supplementary Fig. 3). All four CpG sites showed lower methylation levels in BPD+T compared to BPD-T subjects.

**Table 3:** Top 20 differentially methylated CpG sites resulting from the comparison between BPD+T patients (*N* = 49) and BPD-T patients (*N* = 47).

CpG site	*P* value	*Q* value	*B*	Chr.	Chr. Position	Gene	Gene region
cg01652665	1.25E−06	5.30E−01	3.71	14	36987722	NKX2-1	Body
cg24786705	4.11E−06	8.73E−01	2.55	5	34500318		IGR^a^
cg10888111	6.44E−06	9.11E−01	2.12	2	1637068	PXDN	3′UTR^a^
cg15948871	1.01E−05	9.46E−01	1.68	6	31139620	POU5F1	TSS1500^a^
cg05762616	1.41E−05	9.46E−01	1.35	10	133787150	BNIP3	Body
cg13911052	1.47E−05	9.46E−01	1.32	2	176978909		IGR
cg24846397	1.56E−05	9.46E−01	1.26	10	133787150	CASP7	TSS1500
cg24915915	1.99E−05	9.54E−01	1.02	2	231799064		IGR
cg04511534	2.02E−05	9.54E−01	1.01	17	4463371	GGT6	Body^a^
cg16465769	2.75E−05	0.999985	0.71	6	43266151	SLC22A7	1stExon^a^
cg01862363	3.21E−05	0.999985	0.56	17	8928028	NTN1	Body
cg12575928	3.37E−05	0.999985	0.51	11	22687660	GAS2	TSS1500
cg02481697	3.68E−05	0.999985	0.43	17	8928012	NTN1	Body
cg20989454	3.82E−05	0.999985	0.39	11	613478	IRF7	Body
cg24319547	3.90E−05	0.999985	0.37	6	33168857	SLC39A7	1stExon
cg17207064	4.08E−05	0.999985	0.33	2	17982866		IGR
cg00914033	4.58E−05	0.999985	0.22	10	81664698		IGR
cg27190024	4.82E−05	0.999985	0.17	7	94034528	COL1A2	Body
cg10969178	5.55E−05	0.999985	0.03	6	31838402	SLC44A4	Body
cg06759559	6.37E−05	0.999985	−0.10	1	7028331	CAMTA1	Body

*Q* value indicates FDR corrected *p* values. *B* indicates log-odds that the gene is differentially expressed.

^a^CpGs in two studies: BPD+T vs. BPD-T & CTL and BPD+T vs. BPD-T.

Interestingly, five of the six most significant CpGs findings in the BPD vs. CTL comparison (first analysis) were in the X chromosome whereas no statistically significant associations were detected with X chromosome CpG sites in the other two analyses. Supplementary Figure 4 shows all Manhattan and Quantile–Quantile (Q–Q) plots of DMCs resulting from different comparisons.

GSEA of CpG results, using GO biological processes gene sets, revealed a significant presence of genes involved in neurogenesis, neuron and cell differentiation and cell transport when comparing BPD patients and control individuals (Supplementary Table 2). Similar findings were observed when investigating genes with CpG sites differentially methylated between BPD+T patients vs. CTL and BPD-T individuals, with regulation of cell differentiation and neurogenesis being the top findings (Supplementary Table 3). GSEA analyses of the results from BPD+T vs. BPD-T comparisons revealed a significant presence of DMCs in genes involved in cellular macromolecule location and in extracellular structure organization (Supplementary Table 4). GSEA using the Hallmark database showed a significant number of DMCs in genes involved in early and late oestrogen response and myogenesis when comparing BPD vs. CTL and BPD+T vs. CTL & BPD-T (Supplementary Tables 5 and 6). However, no statistically significant results were observed when analysing the results of comparing BPD+T vs. BPD-T data.

Comparisons between BPD and CTL subjects revealed 33 DMRs, most of them in the chromosome 6 (major histocompatibility complex locus) and in the X chromosome. GSEA of the 33 genes included within these regions, using GO biological processes gene sets, revealed a significant presence of genes involved in antigen processing and presentation, and in immune response (Supplementary Table 7). Analysis of BPD+T patients vs. CTL and BPD-T individuals uncovered 16 DMR regions that were enriched for genes involved in embryonic development and regulation of gliogenesis (Supplementary Table 8). Analyses of DMRs in BPD+T vs. BPD-T revealed 13 regions although no significant gene set enrichment within these regions was observed.

### Replication study

Four hundred and seven individuals (*N* = 293 BPDs, including 147 with childhood trauma information −81 BPD+T and 66 BPD−T−, and *N* = 114 CTL) and 15 CpG sites (top results from analyses 1, 2 and 3 plus 2 CpGs not statistically significant that were amongst the top findings in two comparisons) were included in the replication study. Five of the selected CpGs (*ZNF41* cg22713892; *TAP2* cg20156774; *BNIP3* cg05762616; *GGT6* cg04511534; *SLC22A7* cg16465769) were not suitable for EpiTYPER® analyses and only ten CpGs were studied (Supplementary Table 9). All samples and amplicons passed Quality Control analyses. Only two of the four CpGs investigated were found statistically significant (*PQBP1* cg10030436, *p* = 1.84 × 10^−02^ and *RPL10* cg02871887, *p* = 2.24 × 10^−02^) when comparing BPD and CTL subjects. Nevertheless, the methylation levels in all CpGs investigated were lower in BPD patients than in controls, coinciding with the findings in the discovery study.

The *PXDN CpG* (cg10888111) was found differentially methylated in the BPD+T vs. BPD−T & CTL comparison (*p* = 5.33 × 10^−3^), and in the BPD+T vs. BPD−T comparison (*p* = 3.26 × 10^−2^). BPD+T subjects showed lower methylation levels in both analyses, mirroring the findings of the discovery study. Interestingly, the *PXDN* cg10888111 was also found differentially methylated when comparing BPD and CTL subjects in the replication study (*p* = 2.25 × 10^−4^), although it was not found statistically significant in the EWAS. No other significant association was found in the replication study, although the direction of the epigenomic alterations coincided with that of the discovery study.

## Discussion

The present study investigated methylation patterns in patients with BPDs in comparison with controls and investigated the possible contribution of childhood traumas to the differences observed.

As expected, different methylation patterns were observed in patients when compared to controls, as well as in individuals with childhood trauma and without. The methylation differences observed between BPD patients and controls did not coincide, in general, with the differences observed when analysing childhood trauma. For instance, comparisons between BPD patients and controls revealed several X chromosome regions differentially methylated, an observation that was not replicated when comparing subjects with or without childhood trauma. These X chromosome epigenomic alterations may partially explain the different gender prevalence and presentation of BPD. In support of this hypothesis, gene enrichment analyses also revealed epigenomic alterations in genes controlling oestrogen regulation, although further confirmation of these findings is required. Methylation alterations were also observed in genes involved in neurogenesis, neuron differentiation, development, regulation and morphogenesis, as revealed by the GSEA, whereas DMRs analyses revealed enrichment of genes involved in the regulation of immune response processes.

The discovery study revealed that methylation levels of 5 X chromosome CpG sites within the *PQBP1*, *ZNF41*, *RPL10* genes and two intragenic regions were lower in BPD patients than in controls. Additionally, a CpG site in the transporter 2 member of the ATP-binding cassette subfamily B (*TAP2*, chromosome 6) was also found to be less methylated in BPD patients than in controls. The Polyglutamine binding protein 1 (PQBP1) is involved in transcription activation. *PQBP1* mutations have been linked with intellectual disability and neurodegenerative disorders^42^ and with Renpenning syndrome^43^. The zinc-finger protein 41 (ZNF41) participates in nucleic acid binding and transcription regulation. *ZNF41* mutations have been associated with cognitive deficits^44^. However, this finding was not confirmed in later studies^45^. The ribosomal protein L10 (RPL10) is a component of the 60S ribosomal subunit and participates in RNA binding and transcription and translation regulation. RPL10 dysfunction disrupts neurodevelopment and causes X-linked ribosomopathy characterized by syndromic intellectual disability and epilepsy^46^. RPL10 mutations have been linked to autism spectrum disorder (ASD) mechanisms^47^. Cognitive and personality traits share common genetic factors and symptomatology^48^. For instance, impulsivity and emotional dysregulation are symptoms common to individuals with personality disorders or significant intellectual disability^49^. These epigenetic alterations may partially explain the similarities in symptomatology between BPD, ASD and intellectual disability. Another X chromosome intragenic CpG island (cg24395855) found to be associated with BPD risk is within a region not previously associated with mental disorders or its symptomatology. Less clear is the link observed between BPD risk and low methylation of the TAP2 gene, involved in antigen presentation. Mutations in *TAP2* have been associated with several diseases including cancer, tuberculosis, diabetes and arthritis although no link to mental disorders has been reported.

Interestingly, the differences in methylation levels were bigger when comparing BPD patients with trauma vs. controls than when comparing BPD without trauma vs. controls. These observations suggest that childhood trauma confers epigenomic alterations that modulate or increase BPD presentation and symptomatology.

Epigenome wide comparisons between BPD+T and BPD-T & CTLS revealed significant differences in the level of methylation of CpG islands in five genes, including *POU5F1*, *GPR55*, *GGT6*, *TNFRSF13C*, and *FAM113B*, none of them located in the X chromosome. The methylation levels of these islands were lower in BPD patients with childhood trauma than in BPD patients without childhood trauma and controls. The POU5F1 (OCT4) is a transcription factor that plays a key role in embryonic development and stem cell pluripotency, and it has been linked to several forms of cancer. The *GPR55* gene codes for a putative G-protein-coupled cannabinoid receptor that activates a variety of transduction signal pathways. *GPR55* polymorphisms have been associated with anorexia nervosa^50^, cancer and Crohn’s disease and it has been hypothesized to play a potential role in obesity^51^. No clear links between this receptor and mental disorders has been reported, although in a previous study increased levels of GPR55 were found in mouse models of RETT syndrome^52^. GGT6 is a gamma-glutamyltransferase that plays a key role in glutathione homoeostasis by providing substrates for its synthesis^53^. It has been associated with cancer and rheumatoid arthritis with no links with mental disorders. TNFRSF13C enhances B cell survival and regulates B cell population. Recently, a microdeletion in 22q including this gene was associated with intellectual disability ASD^54^. FAM113B main function is the modification of biopolymers on the cell surface and has been associated with lung cancer^55^. GSA of these results confirmed that childhood trauma altered the methylation levels of genes involved in cell regulation and neurogenesis. Additionally, DMRs analyses revealed enrichment of genes involved in the regulation of gliogenesis and embryonic development.

Although no statistically significant findings were obtained when comparing patients with and without childhood trauma, probably due to the reduced sample size, several interesting associations were observed, including regions in the genes *NKX2-1*, *PXDN* and *POU5F1*. Interestingly, *POU5F1* was also found differentially methylated in this reduced sample, with BPD patients with childhood trauma presenting lower levels than those without trauma, thus replicating the previous finding. NKX2-1 regulates expression of thyroid and morphogenesis genes. Several studies report a clear association between alterations in NKX2-1 and thyroid and lung cancer, although it has also been hypothesized to play a role in the development of schizophrenia through the regulation of implicated pathways. PXDN is a peroxidase involved in external matrix formation associated with obesity risk, ovarian^56^ and prostate cancer^57^, and more interestingly, with ASD^58^. A deletion of a chromosome 2 region including the *PXDN* gene has also been associated with intellectual disability and obesity^59^. However, these two later reports have not been replicated. Although no significant gene enrichment results were obtained when analysing BPD+T and BPD−T, several top findings replicated those found when analysing trauma in the whole cohort (i.e. neurogenesis and cell replication), suggesting that they are true results.

Previous reports have linked methylation levels in the gene *NR3C1*, involved in stress response, to trauma, major depression, post-traumatic stress disorder and personality disorders^60^. In a previous study we reported an association between higher methylation levels in *NR3C1* and BPD risk and childhood trauma^26^, a finding that coincided with those of Perroud and colleagues^27^. Although we did not find statistically significant differences in the *NRC31* methylation sites, we did find a nominal association also showing higher methylation of a *NRC31* CpG site in BPD patients. A previous study had also found increased methylation levels of *5-HT2A*, *MAOA*, *MAOB* and *S-COMT* genes in 26 BPD patients compared with 11 controls^28^. We were not able to replicate these findings, as we detected hypo and hypermethylation sites in all those genes when comparing BPD patients and controls, and none of these differences reached statistical significance. Differences in sample size and sites analysed may explain this discrepancy. Interestingly, an EWAS study performed in BPD patients also found epigenomic alterations in genes involved in cell and neurogenesis regulation associated with the severity of the disorder, with their top finding in the X chromosome^29^. However, their results are not comparable to ours as male and female BPD patients were compared to major depression patients in their study, with no general population controls considered.

Our sample has several limitations. First, the moderate sample size used in the discovery sample hinders the reliability of the results. To minimize false-positive findings, we investigated the top findings in a second larger replication sample. Most of the investigated findings were confirmed in the replication sample, or showed similar directions as in the discovery study, confirming their reliability. Secondly, the controls used in the discovery sample did not experience childhood trauma according to the questionnaires and childhood trauma data were not available in all replication samples. This information might help to infer the contribution of childhood trauma to epigenomic changes. Thirdly, smoking habits were not considered in the analyses, although they are similar in BPD patients and unaffected individuals. Fourthly, we did not have detailed information on comorbidities (i.e. ADHD or affective disorders) nor on pharmacological treatment received by the study subjects. Both issues may hinder the analyses and difficult the obtention of clear results. Additionally, we did not have data on body mass index, socioeconomic status or education and did not include these variables in the analyses. Fourthly, cell-type proportions were not known in the replication sample, so the results of our discovery study may not be entirely reproducible in the replication study. Finally, although we conducted our study on DNA extracted from blood cells instead of brain tissue, there is a high correlation (*r* = 0.86) between blood and brain methylation status^61^.

In summary, we found epigenomic alterations in X chromosome and oestrogen regulation genes that may contribute to the development of BPD and explain, at least partially, the differences in presentation between genders. Furthermore, childhood trauma events modulated the magnitude of the epigenomic alterations contributing to BPD, confirming previous findings.

## Supplementary information

Supplemental Material

## References

[1] Widiger TA, Weissman MM (1991). Epidemiology of borderline personality disorder. Hosp. Community Psychiatry.

[2] Bassir Nia A (2018). Past, present, and future of genetic research in borderline personality disorder. Curr. Opin. Psychol..

[3] Alvarez-Tomás I (2017). Long-term course of borderline personality disorder: a prospective 10-year follow-up study. J. Pers. Disord..

[4] Beauchaine TP, Klein DN, Crowell SE, Derbidge C, Gatzke-Kopp L (2009). Multifinality in the development of personality disorders: a biology x sex x environment interaction model of antisocial and borderline traits. Dev. Psychopathol..

[5] Crowell SE, Beauchaine TP, Linehan MM (2009). A biosocial developmental model of borderline personality: elaborating and extending Linehan’s theory. Psychol. Bull..

[6] Porter C (2020). Childhood adversity and borderline personality disorder: a meta-analysis. Acta Psychiatr. Scand..

[7] Ten Have M (2016). Prevalence rates of borderline personality disorder symptoms: a study based on the Netherlands Mental Health Survey and Incidence Study-2. BMC Psychiatry.

[8] Skoglund, C. et al. Familial risk and heritability of diagnosed borderline personality disorder: a register study of the Swedish population. *Mol. Psychiatry* (2019). 10.1038/s41380-019-0442-0 PMC7910208 31160693

[9] Sansone RA, Sansone LA (2011). Gender patterns in borderline personality disorder. Innov. Clin. Neurosci..

[10] Amad A, Ramoz N, Thomas P, Jardri R, Gorwood P (2014). Genetics of borderline personality disorder: systematic review and proposal of an integrative model. Neurosci. Biobehav. Rev..

[11] Martín-Blanco A (2016). The role of hypothalamus-pituitary-adrenal genes and childhood trauma in borderline personality disorder. Eur. Arch. Psychiatry Clin. Neurosci..

[12] Witt SH (2017). Genome-wide association study of borderline personality disorder reveals genetic overlap with bipolar disorder, major depression and schizophrenia. Transl. Psychiatry.

[13] Martín-Blanco A (2015). An exploratory association study of the influence of noradrenergic genes and childhood trauma in borderline personality disorder. Psychiatry Res..

[14] Amad A, Ramoz N, Peyre H, Thomas P, Gorwood P (2019). FKBP5 gene variants and borderline personality disorder. J. Affect. Disord..

[15] Thomas M (2018). Increased BDNF methylation in saliva, but not blood, of patients with borderline personality disorder. Clin. Epigenet..

[16] Bockmühl Y (2015). Methylation at the CpG island shore region upregulates Nr3c1 promoter activity after early-life stress. Epigenetics.

[17] Perroud N (2014). Childhood maltreatment and methylation of the glucocorticoid receptor gene NR3C1 in bipolar disorder. Br. J. Psychiatry.

[18] Thaler L (2014). Methylation of BDNF in women with bulimic eating syndromes: associations with childhood abuse and borderline personality disorder. Prog. Neuropsychopharmacol. Biol. Psychiatry.

[19] Tyrka AR (2015). Childhood maltreatment and methylation of FK506 binding protein 5 gene (FKBP5). Dev. Psychopathol..

[20] Marinova Z, Maercker A, Grünblatt E, Wojdacz TK, Walitza S (2017). A pilot investigation on DNA methylation modifications associated with complex posttraumatic symptoms in elderly traumatized in childhood. BMC Res. Notes.

[21] Nestler EJ, Peña CJ, Kundakovic M, Mitchell A, Akbarian S (2016). Epigenetic basis of mental illness. Neuroscientist.

[22] Wolf EJ (2018). Traumatic stress and accelerated DNA methylation age: a meta-analysis. Psychoneuroendocrinology.

[23] Kuehner JN, Bruggeman EC, Wen Z, Yao B (2019). Epigenetic regulations in neuropsychiatric disorders. Front. Genet.

[24] Sumner JA, Colich NL, Uddin M, Armstrong D, McLaughlin KA (2019). Early experiences of threat, but not deprivation, are associated with accelerated biological aging in children and adolescents. Biol. Psychiatry.

[25] Bustamante AC, Armstrong DL, Uddin M (2018). Epigenetic profiles associated with major depression in the human brain. Psychiatry Res..

[26] Martín-Blanco A (2014). Association between methylation of the glucocorticoid receptor gene, childhood maltreatment, and clinical severity in borderline personality disorder. J. Psychiatr. Res..

[27] Perroud N (2011). Increased methylation of glucocorticoid receptor gene (NR3C1) in adults with a history of childhood maltreatment: a link with the severity and type of trauma. Transl. Psychiatry.

[28] Dammann G (2011). Increased DNA methylation of neuropsychiatric genes occurs in borderline personality disorder. Epigenetics.

[29] Prados J (2015). Borderline personality disorder and childhood maltreatment: a genome-wide methylation analysis. Genes Brain Behav..

[30] First, M., Gibbon, M., Spitzer, R., Benjamin, L. *User’ Guide for the Structured Clinical Interview for DSM-IV Axis II Personality Disorders: SCID-II* (American Psychiatric Publishing, 1997).

[31] Barrachina J (2011). Axis II comorbidity in borderline personality disorder is influenced by sex, age, and clinical severity. Compr. Psychiatry.

[32] Bernstein DP (2003). Development and validation of a brief screening version of the Childhood Trauma Questionnaire. Child Abuse Negl..

[33] Touleimat N, Tost J (2012). Complete pipeline for Infinium(®) Human Methylation 450K BeadChip data processing using subset quantile normalization for accurate DNA methylation estimation. Epigenomics.

[34] Pidsley R (2013). A data-driven approach to preprocessing Illumina 450K methylation array data. BMC Genomics.

[35] Zhou W, Laird PW, Shen H (2017). Comprehensive characterization, annotation and innovative use of Infinium DNA methylation BeadChip probes. Nucleic Acids Res..

[36] Tian Y (2017). ChAMP: updated methylation analysis pipeline for Illumina BeadChips. Bioinformatics.

[37] Houseman EA (2012). DNA methylation arrays as surrogate measures of cell mixture distribution. BMC Bioinformatics.

[38] Johnson WE, Li C, Rabinovic A (2007). Adjusting batch effects in microarray expression data using empirical Bayes methods. Biostatistics.

[39] Ritchie ME (2015). limma powers differential expression analyses for RNA-sequencing and microarray studies. Nucleic Acids Res.

[40] Rakyan VK, Down TA, Balding DJ, Beck S (2011). Epigenome-wide association studies for common human diseases. Nat. Rev. Genet..

[41] Liberzon A (2011). Molecular signatures database (MSigDB) 3.0. Bioinformatics.

[42] Okazawa H (2018). PQBP1, an intrinsically disordered/denatured protein at the crossroad of intellectual disability and neurodegenerative diseases. Neurochem Int..

[43] Jeong HI (2018). Ann. Clin. Lab. Sci..

[44] Shoichet SA (2003). Mutations in the ZNF41 gene are associated with cognitive deficits: identification of a new candidate for X-linked mental retardation. Am. J. Hum. Genet..

[45] Piton A, Redin C, Mandel JL (2013). XLID-causing mutations and associated genes challenged in light of data from large-scale human exome sequencing. Am. J. Hum. Genet..

[46] Bourque DK (2018). A de novo mutation in RPL10 causes a rare X-linked ribosomopathy characterized by syndromic intellectual disability and epilepsy: A new case and review of the literature. Eur. J. Med. Genet..

[47] Klauck SM (2006). Mutations in the ribosomal protein gene RPL10 suggest a novel modulating disease mechanism for autism. Mol. Psychiatry.

[48] Briley DA, Tucker-Drob EM (2017). Comparing the developmental genetics of cognition and personality over the life span. J. Pers..

[49] Cowan, A. Too many feelings: a case series of individuals with borderline personality disorders and intellectual disability. *J. Child Dev. Disord.* **4**, 10.4172/2472-1786.100072 (2018).

[50] Ishiguro H (2011). Functional polymorphism in the GPR55 gene is associated with anorexia nervosa. Synapse.

[51] Moreno-Navarrete JM (2012). The L-α-lysophosphatidylinositol/GPR55 system and its potential role in human obesity. Diabetes.

[52] Vigli D (2018). Chronic treatment with the phytocannabinoid Cannabidivarin (CBDV) rescues behavioural alterations and brain atrophy in a mouse model of Rett syndrome. Neuropharmacology.

[53] Heisterkamp N, Groffen J, Warburton D, Sneddon TP (2008). The human gamma-glutamyltransferase gene family. Hum. Genet.

[54] Upadia J (2018). A previously unrecognized 22q13.2 microdeletion syndrome that encompasses TCF20 and TNFRSF13C. Am. J. Med. Genet. A.

[55] Hong Y. et al. Epigenome-wide association analysis of differentially methylated signals in blood samples of patients with non-small-cell lung cancer. *J. Clin. Med.* **8**, 1307 (2019). 10.3390/jcm8091307 PMC6780065 31450665

[56] Zheng YZ, Liang L (2018). High expression of PXDN is associated with poor prognosis and promotes proliferation, invasion as well as migration in ovarian cancer. Ann. Diagn. Pathol..

[57] Dougan, J. et al. Proteomics-metabolomics combined approach identifies peroxidasin as a protector against metabolic and oxidative stress in prostate cancer. *Int. J. Mol. Sci.* **20**, 3046 (2019). 10.3390/ijms20123046. 10.3390/ijms20123046 PMC6627806 31234468

[58] Meyer KJ, Axelsen MS, Sheffield VC, Patil SR, Wassink TH (2012). Germline mosaic transmission of a novel duplication of PXDN and MYT1L to two male half-siblings with autism. Psychiatr. Genet..

[59] Bonaglia MC, Giorda R, Zanini S (2014). A new patient with a terminal de novo 2p25.3 deletion of 1.9 Mb associated with early-onset of obesity, intellectual disabilities and hyperkinetic disorder. Mol. Cytogenet.

[60] Watkeys OJ, Kremerskothen K, Quidé Y, Fullerton JM, Green MJ (2018). Glucocorticoid receptor gene (NR3C1) DNA methylation in association with trauma, psychopathology, transcript expression, or genotypic variation: a systematic review. Neurosci. Biobehav. Rev..

[61] Braun PR (2019). Genome-wide DNA methylation comparison between live human brain and peripheral tissues within individuals. Transl. Psychiatry.

