# Supplementary material for: A genome-wide methylation study reveals X chromosome and childhood trauma methylation alterations associated with borderline personality disorder
Source: Transl Psychiatry. 2021 Jan 5;11:5. doi: 10.1038/s41398-020-01139-z (PMC7791113; doi:10.1038/s41398-020-01139-z)

**Supplemental Material**

**Tables and Figures**

**Supplementary Table 1**. Demographic, Clinical and CTQ-SF data of study cohorts.

| **Variables** | | **BPD**  **(N=147)** | | **BPD+T**  **(N=81)** | | **BPD-T**  **(N=66)** | | **p** |
| --- | --- | --- | --- | --- | --- | --- | --- | --- |
| Demographic |  | |  | |  | |  |  |
| Age [mean (SD)] | | 30.93 (7.2) | | 31.8 (6.9) | | 29.8 (7.3) | | n.s |
| Clinical |  | |  | |  | |  |  |
| DIB-R, total score [mean (SD)] | | 7.55 (1.3) | | 7.7 (1.3) | | 7.4 (1.3) | | n.s |
| Self-injury [n (%)] | | 79 (53.7%) | | 48 (59.3%) | | 31 (47%) | | n.s |
| Hospitalizations [n (%)] | | 73 (49.7%) | | 32 (39.5%) | | 32 (48.5%) | | n.s |
| CTQ-SF subscales |  | |  | |  | |  |  |
| Emotional abuse | | 86 (58.5%) | | 72 (88.9%) | | 14 (16.3%) | | <.001 |
| Physical abuse | | 46 (31.3%) | | 46 (56.8%) | | 0 (0%) | | <.001 |
| Sexual abuse | | 59 (40.1%) | | 49 (60.5%) | | 10 (15.2%) | | <.001 |
| Emotional negligence | | 71 (48.3%) | | 64 (79.0%) | | 7 (10.6%) | | <.001 |
| Physical negligence | | 40 (27.2%) | | 39 (48.1%) | | 1 (1.5%) | | <.001 |

Note. SD = Standard Deviation. DIB-R = Revised Diagnostic Interview for Borderlines. CTQ-SF = *Childhood Trauma Questionnaire - Short Form.*

**Supplementary Table 2**: Top 20 most significant findings resulting from GO biological processes comparisons with the BPD vs CTL CpG results. q-values indicate FRD corrected p-values.

| **Gene Set Name** | **# Genes in GO  Gene Set** | **# Genes in  Overlap** | **p-value** | **q-value** |
| --- | --- | --- | --- | --- |
| GO_NEUROGENESIS | 1599 | 91 | 5.84E-21 | 4.29E-17 |
| GO_REGULATION_OF_CELL_DIFFERENTIATION | 1863 | 99 | 1.18E-20 | 4.33E-17 |
| GO_REGULATION_OF_TRANSPORT | 1842 | 97 | 5.67E-20 | 1.39E-16 |
| GO_INTRACELLULAR_TRANSPORT | 1825 | 96 | 9.73E-20 | 1.79E-16 |
| GO_NEURON_DIFFERENTIATION | 1348 | 80 | 1.46E-19 | 2.15E-16 |
| GO_CELL_CELL_SIGNALING | 1644 | 89 | 4.08E-19 | 5.00E-16 |
| GO_POSITIVE_REGULATION_OF_MOLECULAR_FUNCTION | 1756 | 91 | 2.56E-18 | 2.69E-15 |
| GO_POSITIVE_REGULATION_OF_BIOSYNTHETIC_PROCESS | 1983 | 97 | 7.95E-18 | 7.30E-15 |
| GO_RESPONSE_TO_OXYGEN_CONTAINING_COMPOUND | 1616 | 84 | 4.96E-17 | 3.78E-14 |
| GO_POSITIVE_REGULATION_OF_GENE_EXPRESSION | 1974 | 95 | 5.15E-17 | 3.78E-14 |
| GO_HOMEOSTATIC_PROCESS | 1913 | 93 | 6.10E-17 | 4.07E-14 |
| GO_REGULATION_OF_NERVOUS_SYSTEM_DEVELOPMENT | 914 | 60 | 7.14E-17 | 4.37E-14 |
| GO_NEURON_DEVELOPMENT | 1096 | 66 | 1.36E-16 | 7.70E-14 |
| GO_POSITIVE_REGULATION_OF_RNA_BIOSYNTHETIC_PROCESS | 1600 | 82 | 2.72E-16 | 1.43E-13 |
| GO_REGULATION_OF_CELLULAR_LOCALIZATION | 886 | 57 | 1.06E-15 | 5.11E-13 |
| GO_CELLULAR_COMPONENT_MORPHOGENESIS | 1115 | 65 | 1.11E-15 | 5.11E-13 |
| GO_INTERSPECIES_INTERACTION_BETWEEN_ORGANISMS | 927 | 58 | 2.00E-15 | 8.65E-13 |
| GO_CELLULAR_MACROMOLECULE_LOCALIZATION | 1897 | 89 | 2.53E-15 | 1.03E-12 |
| GO_REGULATION_OF_CELL_DEVELOPMENT | 939 | 58 | 3.50E-15 | 1.35E-12 |

**Supplementary Table 3**: Top 20 most significant findings resulting from GO biological processes comparisons with the BPD+T vs BPD-T & CTL CpG results. q-values indicate FRD corrected p-values.

| **Gene Set Name** | **# Genes in GO  Gene Set** | **# Genes in  Overlap** | **p-value** | **q-value** |
| --- | --- | --- | --- | --- |
| GO_REGULATION_OF_CELL_DIFFERENTIATION | 1863 | 150 | 3.66E-32 | 2.69E-28 |
| GO_NEUROGENESIS | 1599 | 129 | 1.05E-27 | 3.87E-24 |
| GO_BIOLOGICAL_ADHESION | 1417 | 118 | 1.52E-26 | 3.72E-23 |
| GO_NEURON_DIFFERENTIATION | 1348 | 107 | 1.71E-22 | 3.15E-19 |
| GO_CELLULAR_MACROMOLECULE_LOCALIZATION | 1897 | 130 | 1.64E-21 | 2.41E-18 |
| GO_POSITIVE_REGULATION_OF_MULTICELLULAR_ORGANISMAL_PROCESS | 1795 | 125 | 2.82E-21 | 3.46E-18 |
| GO_POSITIVE_REGULATION_OF_DEVELOPMENTAL_PROCESS | 1415 | 107 | 6.72E-21 | 7.06E-18 |
| GO_POSITIVE_REGULATION_OF_BIOSYNTHETIC_PROCESS | 1983 | 132 | 9.57E-21 | 8.38E-18 |
| GO_REGULATION_OF_INTRACELLULAR_SIGNAL_TRANSDUCTION | 1846 | 126 | 1.03E-20 | 8.38E-18 |
| GO_ANIMAL_ORGAN_MORPHOGENESIS | 1034 | 88 | 1.43E-20 | 1.05E-17 |
| GO_POSITIVE_REGULATION_OF_GENE_EXPRESSION | 1974 | 129 | 1.29E-19 | 8.65E-17 |
| GO_REGULATION_OF_CELL_POPULATION_PROLIFERATION | 1708 | 117 | 2.18E-19 | 1.28E-16 |
| GO_NEGATIVE_REGULATION_OF_RNA_BIOSYNTHETIC_PROCESS | 1246 | 96 | 2.26E-19 | 1.28E-16 |
| GO_CELL_CELL_SIGNALING | 1644 | 114 | 2.63E-19 | 1.28E-16 |
| GO_POSITIVE_REGULATION_OF_SIGNALING | 1828 | 122 | 2.72E-19 | 1.28E-16 |
| GO_POSITIVE_REGULATION_OF_RNA_BIOSYNTHETIC_PROCESS | 1600 | 112 | 2.78E-19 | 1.28E-16 |
| GO_NEGATIVE_REGULATION_OF_BIOSYNTHETIC_PROCESS | 1631 | 113 | 4.10E-19 | 1.77E-16 |
| GO_INTRACELLULAR_TRANSPORT | 1825 | 121 | 6.49E-19 | 2.65E-16 |
| GO_CELLULAR_COMPONENT_MORPHOGENESIS | 1115 | 88 | 1.76E-18 | 6.65E-16 |

**Supplementary Table 4**: Top 20 most significant findings resulting from GO biological processes comparisons with the BPD+T vs BPD-T CpG results. q-values indicate FRD corrected p-values.

| **Gene Set Name** | **# Genes in GO  Gene Set** | **# Genes in  Overlap** | **p-value** | **q-value** |
| --- | --- | --- | --- | --- |
| GO_CELLULAR_MACROMOLECULE_LOCALIZATION | 1897 | 29 | 1.29E-07 | 9.51E-04 |
| GO_EXTRACELLULAR_STRUCTURE_ORGANIZATION | 421 | 13 | 2.98E-07 | 9.96E-04 |
| GO_CATION_TRANSMEMBRANE_TRANSPORT | 847 | 18 | 4.07E-07 | 9.96E-04 |
| GO_BIOLOGICAL_ADHESION | 1417 | 23 | 1.07E-06 | 1.69E-03 |
| GO_CELL_CELL_SIGNALING | 1644 | 25 | 1.15E-06 | 1.69E-03 |
| GO_ION_TRANSPORT | 1677 | 25 | 1.64E-06 | 1.81E-03 |
| GO_ION_TRANSMEMBRANE_TRANSPORT | 1138 | 20 | 1.73E-06 | 1.81E-03 |
| GO_CATION_TRANSPORT | 1159 | 20 | 2.28E-06 | 1.96E-03 |
| GO_NEUROGENESIS | 1599 | 24 | 2.40E-06 | 1.96E-03 |
| GO_CELL_CELL_ADHESION | 827 | 16 | 6.03E-06 | 4.28E-03 |
| GO_ANIMAL_ORGAN_MORPHOGENESIS | 1034 | 18 | 6.65E-06 | 4.28E-03 |
| GO_TRANSMEMBRANE_TRANSPORT | 1587 | 23 | 6.99E-06 | 4.28E-03 |
| GO_DEVELOPMENTAL_GROWTH | 663 | 14 | 8.88E-06 | 5.02E-03 |
| GO_CENTRAL_NERVOUS_SYSTEM_DEVELOPMENT | 980 | 17 | 1.27E-05 | 6.66E-03 |
| GO_GROWTH | 990 | 17 | 1.44E-05 | 7.07E-03 |
| GO_INORGANIC_ION_TRANSMEMBRANE_TRANSPORT | 834 | 15 | 2.76E-05 | 1.18E-02 |
| GO_POSITIVE_REGULATION_OF_BIOSYNTHETIC_PROCESS | 1983 | 25 | 2.88E-05 | 1.18E-02 |
| GO_FOREBRAIN_DEVELOPMENT | 381 | 10 | 2.90E-05 | 1.18E-02 |
| GO_EPITHELIUM_DEVELOPMENT | 1278 | 19 | 3.27E-05 | 1.27E-02 |

**Supplementary Table 5**: Top 10 most significant findings resulting from Hallmark gene enrichment analyses with the BPD vs CTL CpG results. q-values indicate FRD corrected p-values.

| **Gene Set Name** | **# Genes in Hallmark  Gene Set** | **# Genes in  Overlap** | **p-value** | **q-value** |
| --- | --- | --- | --- | --- |
| HALLMARK_ESTROGEN_RESPONSE_EARLY | 200 | 15 | 6.71E-06 | 1.68E-04 |
| HALLMARK_IL2_STAT5_SIGNALING | 200 | 15 | 6.71E-06 | 1.68E-04 |
| HALLMARK_MYOGENESIS | 200 | 13 | 1.18E-04 | 1.97E-03 |
| HALLMARK_UV_RESPONSE_DN | 144 | 10 | 4.19E-04 | 3.69E-03 |
| HALLMARK_ALLOGRAFT_REJECTION | 200 | 12 | 4.43E-04 | 3.69E-03 |
| HALLMARK_KRAS_SIGNALING_UP | 200 | 12 | 4.43E-04 | 3.69E-03 |
| HALLMARK_APICAL_SURFACE | 44 | 5 | 1.38E-03 | 7.65E-03 |
| HALLMARK_MITOTIC_SPINDLE | 199 | 11 | 1.47E-03 | 7.65E-03 |
| HALLMARK_APICAL_JUNCTION | 200 | 11 | 1.53E-03 | 7.65E-03 |
| HALLMARK_INFLAMMATORY_RESPONSE | 200 | 11 | 1.53E-03 | 7.65E-03 |

**Supplementary Table 6**: Top 10 most significant findings resulting from Hallmark gene enrichment analyses with the BPD+T vs BPD-T & CTL CpG results. q-values indicate FRD corrected p-values.

| **Gene Set Name** | **# Genes in Hallmark  Gene Set** | **# Genes in  Overlap** | **p-value** | **q-value** |
| --- | --- | --- | --- | --- |
| HALLMARK_ESTROGEN_RESPONSE_EARLY | 200 | 19 | 3.51E-06 | 5.85E-05 |
| HALLMARK_ESTROGEN_RESPONSE_LATE | 200 | 19 | 3.51E-06 | 5.85E-05 |
| HALLMARK_MYOGENESIS | 200 | 19 | 3.51E-06 | 5.85E-05 |
| HALLMARK_HYPOXIA | 200 | 18 | 1.33E-05 | 1.66E-04 |
| HALLMARK_ADIPOGENESIS | 200 | 17 | 4.73E-05 | 4.73E-04 |
| HALLMARK_ALLOGRAFT_REJECTION | 200 | 16 | 1.59E-04 | 1.02E-03 |
| HALLMARK_OXIDATIVE_PHOSPHORYLATION | 200 | 16 | 1.59E-04 | 1.02E-03 |
| HALLMARK_PI3K_AKT_MTOR_SIGNALING | 105 | 11 | 1.64E-04 | 1.02E-03 |
| HALLMARK_MITOTIC_SPINDLE | 199 | 15 | 4.74E-04 | 2.08E-03 |
| HALLMARK_EPITHELIAL_MESENCHYMAL_TRANSITION | 200 | 15 | 5.00E-04 | 2.08E-03 |

**Supplementary Table 7**: Top significant findings resulting from GO biological processes comparisons with the BPD vs CTL DMRs results. q-values indicate FRD corrected p-values.

| **Gene Set Name** | **# Genes in GO  Gene Set** | **# Genes in  Overlap** | **p-value** | **q-value** |
| --- | --- | --- | --- | --- |
| GO_ANTIGEN_PROCESSING_AND_PRESENTATION_OF_PEPTIDE_ANTIGEN | 190 | 5 | 5.08E-07 | 3.83E-03 |
| GO_ANTIGEN_PROCESSING_AND_PRESENTATION | 227 | 5 | 1.22E-06 | 4.60E-03 |
| GO_DEFENSE_RESPONSE | 1765 | 9 | 9.61E-06 | 2.41E-02 |
| GO_T_CELL_RECEPTOR_SIGNALING_PATHWAY | 201 | 4 | 2.33E-05 | 4.39E-02 |
| GO_RESPONSE_TO_BIOTIC_STIMULUS | 1567 | 8 | 3.30E-05 | 4.56E-02 |
| GO_CYTOKINE_MEDIATED_SIGNALING_PATHWAY | 791 | 6 | 4.30E-05 | 4.56E-02 |
| GO_POSITIVE_REGULATION_OF_IMMUNE_SYSTEM_PROCESS | 1187 | 7 | 4.53E-05 | 4.56E-02 |
| GO_DEFENSE_RESPONSE_TO_OTHER_ORGANISM | 1200 | 7 | 4.85E-05 | 4.56E-02 |
| GO_INTERFERON_GAMMA_MEDIATED_SIGNALING_PATHWAY | 88 | 3 | 5.50E-05 | 4.60E-02 |

**Supplementary Table 8**: Top significant findings resulting from GO biological processes comparisons with the BPD vs CTL DMRs results. q-values indicate FRD corrected p-values.

| **Gene Set Name** | **# Genes in GO  Gene Set** | **# Genes in  Overlap** | **p-value** | **q-value** |
| --- | --- | --- | --- | --- |
| GO_EMBRYONIC_ORGAN_DEVELOPMENT | 434 | 5 | 3.32E-07 | 2.50E-03 |
| GO_INTERFERON_GAMMA_MEDIATED_SIGNALING_PATHWAY | 88 | 3 | 4.16E-06 | 1.56E-02 |
| GO_REGULATION_OF_GLIOGENESIS | 124 | 3 | 1.17E-05 | 2.92E-02 |
| GO_EMBRYO_DEVELOPMENT | 1029 | 5 | 2.24E-05 | 4.21E-02 |

**Supplementary Table 9**. CpG sites selected for the replication study. List of 10 top CpG sites suitable to be analyzed via EpiTYPER^®^. Annotation and amplicon information for each CpGs is provided: chromosome (Chr), gene, CpGs position, number of CpG sites in the amplicon, CpG Units assessed per amplicon and UCSC annotation.

| **CpG site** | **Chr** | **Gene** | **CpG Unit** | **CpG in  amplicon** | **CpG Units  studied** | **UCSC annotation  (hg19)** |
| --- | --- | --- | --- | --- | --- | --- |
| cg10888111 | 2 | *PXDN* | JCP_25_CpG_8 | 247 | 5 | chr2:1637068-1637069 |
| cg24915915 | 2 | *GPR55* | JCP_13_CpG_2 | 54 | 3 | chr2:231799064-231799065 |
| cg24786705 | 5 | *-* | JCP_16_CpG_6 | 298 | 5 | chr5:34500318-34500319 |
| cg15948871 | 6 | *POU5F1* | JCP_15_CpG_2 | 53 | 3 | chr6:31139620-31139621 |
| cg05478172 | 12 | *FAM113B* | JCP_26_CpG_18 | 265 | 11 | chr12:47628981-47628982 |
| cg01652665 | 14 | *NKX2-1/SFTA3* | JCP_12_CpG_1 | 36 | 7 | chr14:36987722-36987723 |
| cg02871887 | X | *RPL10* | JCP_20_CpG_34 | 358 | 11 | chrX:153626455-153626456 |
| cg07810091 | X | *-* | JCP_34_CpG_4 | 89 | 9 | chrX:48534493-48534494 |
| cg08065501 | X | *PQBP1* | JCP_33_CpG_6 | 151 | 4 | chrX:48755716-48755717 |
| cg24395855 | X | *-* | JCP_21_CpG_2 | 115 | 4 | chrX:153575581-153575582 |

**Supplementary Figure 1**: Significantly differentially methylated CpG sites resulting from the comparison between BPD (N=96) and CTL (N=44). Data are represented by subgroups: BPD+T (brown), BPD-T (yellow) and CTL (blue). Y-axis represents methylation levels (0= not methylated, 1= fully methylated).


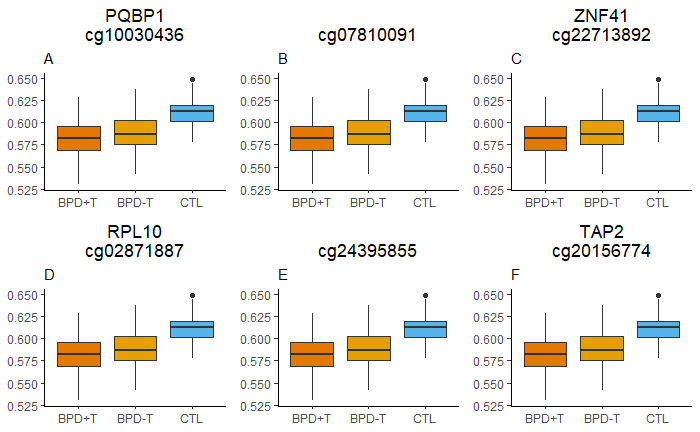


**Supplementary Figure 2**: Significantly differentially methylated CpG sites resulting from the comparison between BPD+T (N=49, in yellow) and BPD-T & CTL (N=91, in blue). Y-axis represents methylation levels (0= not methylated, 1= fully methylated).


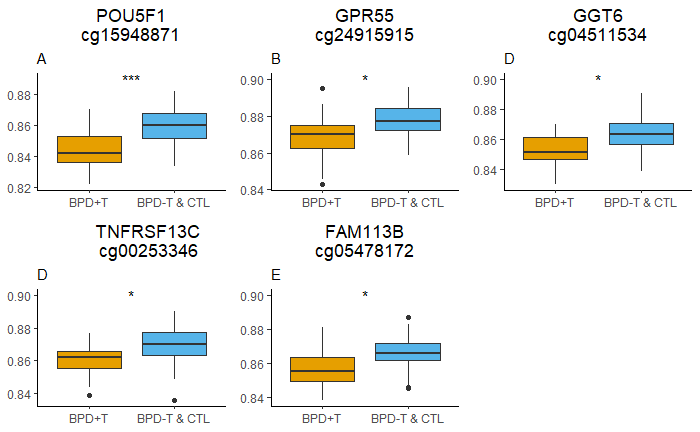


**Supplementary Figure 3**: Top differentially methylated CpG sites resulting from the comparison between BPD+T (N= 49, in yellow) vs BPD-T (N= 47, in yellow). Y-axis represents methylation levels (0= not methylated, 1= fully methylated).


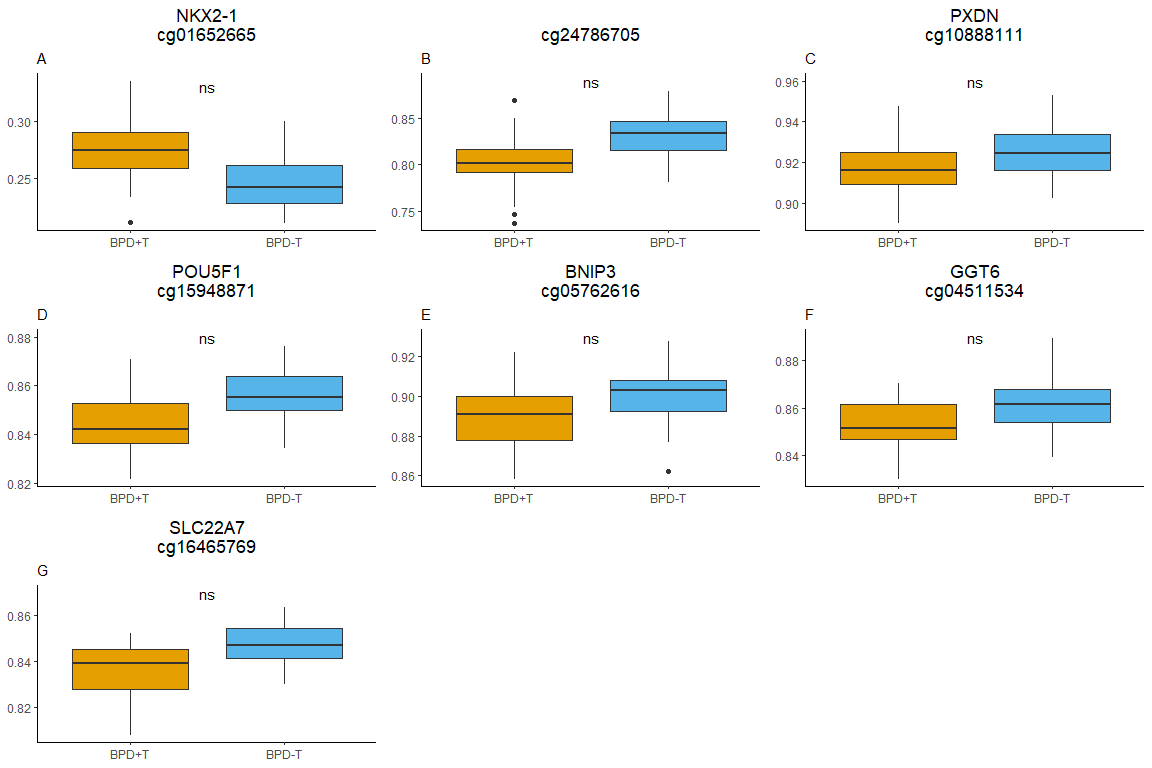


**Supplementary Figure 4:** Manhattan plot and q-q plot of differentially methylated CpG sites resulting from the comparison between A-B) BPD (N=96) and CTL (N=44); C-D) BPD+T (N=49) and BPD-T & CTL (N=91); E-F) BPD+T (N= 49) vs BPD-T (N= 47). In the Manhattan plots, x-axe represents the CpGs position and y-axe the -log10 p-value. Significative threshold line for p-values is in red (10-07), and suggestive line is in blue. For q-q plots, x-axe represents expected -log10 p-values and y-axe observed -log10 p-values. Lambda (λ) is the genomic inflation factor.


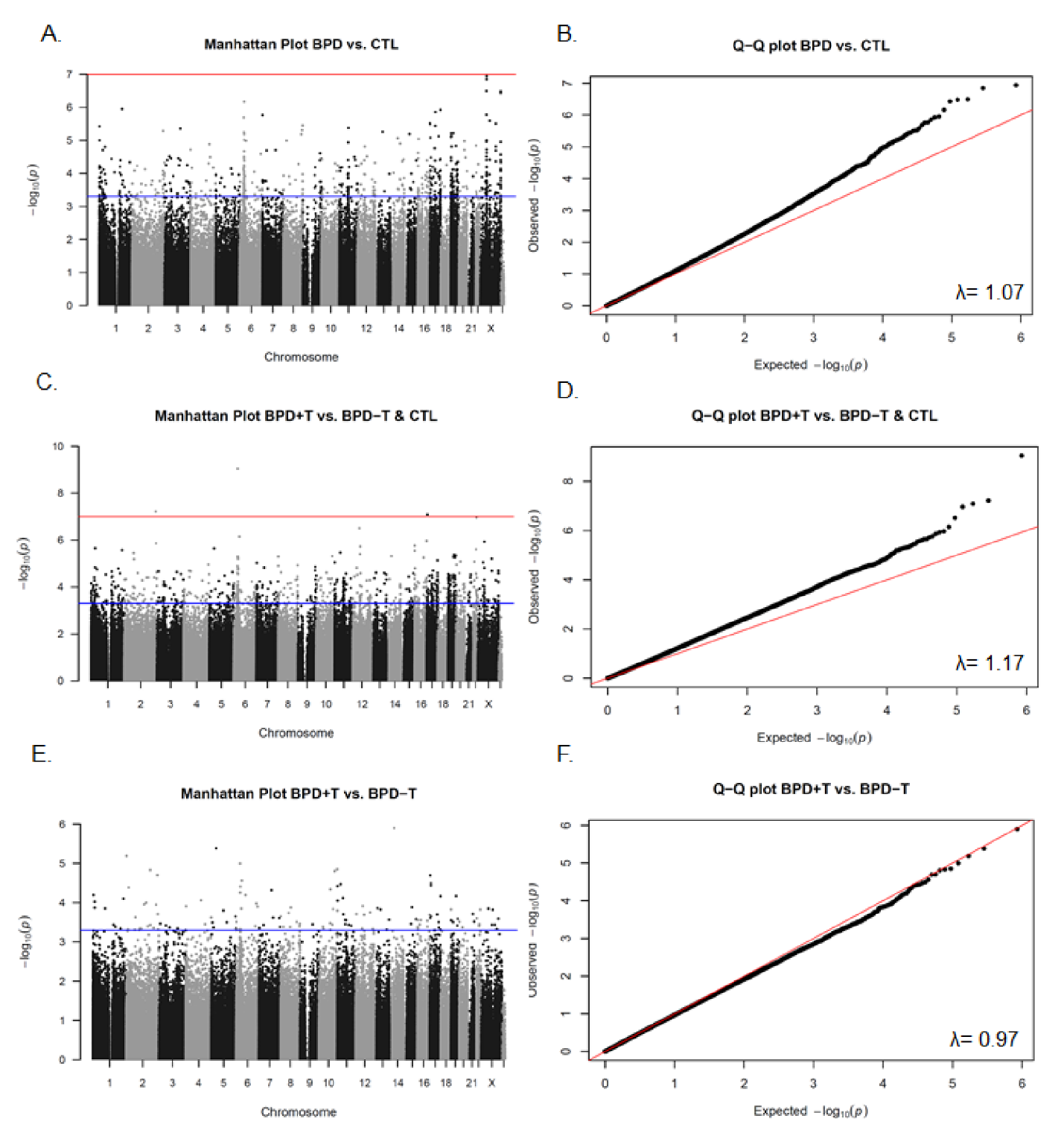

Supplement: Supplementary file 1 — Supplemental Material [file 41398_2020_1139_MOESM1_ESM.docx]
